# Arctigenin inhibits prostate tumor growth in high-fat diet fed mice through dual actions on adipose tissue and tumor

**DOI:** 10.1038/s41598-020-58354-3

**Published:** 2020-01-29

**Authors:** Qiongyu Hao, Tanya Diaz, Alejandro del Rio Verduzco, Clara E. Magyar, Jin Zhong, Yahya Elshimali, Matthew B. Rettig, Susanne M. Henning, Jaydutt V. Vadgama, Piwen Wang

**Affiliations:** 1 Division of Cancer Research and Training, Charles R. Drew University of Medicine and Science, Los Angeles, CA 90059 USA; 2 Department of Pathology, University of California, Los Angeles, CA 90095 USA; 3 Departments of Medicine and Urology, University of California, Los Angeles, CA 90095 USA; 4 Center for Human Nutrition, University of California, Los Angeles, CA 90095 USA; 5 David Geffen School of Medicine, University of California, Los Angeles, CA 90095 USA; 6 VA Greater Los Angeles Healthcare System, Los Angeles, CA 90073 USA; 7 University of California, Riverside, CA 92521 USA

**Keywords:** Prostate cancer, Prostate

## Abstract

This study investigated the inhibitory effect of arctigenin, a novel anti-inflammatory lignan, on prostate cancer in obese conditions both *in vitro* and *in vivo*. *In vitro* obese models were established by co-culture of mouse adipocytes 3T3-L1 with androgen-sensitive LNCaP human prostate cancer cells, or by culturing LNCaP cells in adipocytes-conditioned medium. Arctigenin significantly inhibited LNCaP proliferation, along with decreased androgen receptor (AR) and increased Nkx3.1 cellular expression. Male severe combined immunodeficiency mice were subcutaneously implanted with human prostate cancer LAPC-4 xenograft tumors for *in vivo* study. Mice were fed high-fat (HF) diet and orally given arctigenin at 50 mg/kg body weight daily or vehicle control for 6 weeks. Tumor bearing HF control mice showed a significant increase in serum free fatty acids (FFAs) and decrease in subcutaneous/peritoneal fat depots compared to non-tumor bearing control mice. Arctigenin intervention significantly reduced tumor growth by 45%, associated with decreased circulating FFAs and adipokines/cytokines including IGF-1, VEGF, and MCP-1, along with decreased AR, Ki67, and microvessel density and increased Nkx3.1 expression in tumors. These results indicate the strong ability of arctigenin to co-target obesity and tumor itself in inhibition of prostate tumor growth at a lower concentration compared to most phytochemicals.

## Introduction

Prostate cancer is the most common type of cancer among men in the United States, and it is the second-leading cause of cancer death among American men^1^. Most prostate tumors grow slowly with a relatively long period before lesions are clinically detectable, and may not cause symptoms until death^2^. However, some cases of prostate cancer are aggressive spreading to other sites rapidly, such as bone and lymph node^3^. It remains a challenge to predict the aggressiveness of prostate cancer, as a result many patients may be over-treated in clinic with surgery and/or antiandrogen, suffering side effects of these therapies like incontinence and impotence^4^. Continuous effort has been made to develop strong while less-toxic approaches in prevention and treatment of prostate cancer.

The tumor-promoting effect of obesity has been widely observed in different types of cancer including prostate cancer, associated with elevated circulating levels of free fatty acid (FFA) and multiple adipokines/cytokines which promote the progression and aggressiveness of cancer cells and induce resistance^5–7^. Obesity is increasingly prevalent that affects more than 30% adults in the United States, and becomes a major health problem worldwide^8^. Obesity is associated with multiple chronic diseases, including coronary artery disease, diabetes, and cancer^5^. Evidence has been consistent that obesity is linked to high-grade prostate cancer and dying from prostate cancer^6,9^. Therefore, it would be necessary to co-target obesity in treatment of prostate cancer particularly in obese patients.

Arctigenin is a lignan compound mainly from the seeds of *Arctium lappa* (also called greater burdock). The root of *Arctium lappa*, which also contains arctigenin, is a popular vegetable and food in many countries including China and Japan^7^. Arctigenin is also found in many other plants such as *Bardanae fructus*, *Saussurea medusa*, and *Torreya nucifera*^10^. Arctigenin exists in plants mainly in its glucoside form, arctiin, which releases arctigenin during the digestive process. After oral administration of arctiin (25–100 mg/kg b.w.) to rats both arctiin and arctigenin were detected in blood (*C*_*max*_: 0.7–2.5 mg/L and 0.23–0.65 mg/L, respectively), and both widely distributed in different tissues including small intestine, stomach, lung and kidney, with concentrations up to 25–30 µg/g tissue^11^. To our knowledge, there are no human studies reported on pure form of arctigenin to date. The anti-carcinogenic activity of arctigenin has been demonstrated in different preclinical cancer models, including pancreatic, breast, and lung cancer. Arctigenin inhibited tumor growth through its ability in regulating cancer cell apoptosis and proliferation through the modulation of multiple signaling pathways^12–14^. We previously found in a xenograft mouse model that arctigenin was a potent inhibitor of prostate tumor growth in non-obese state^15^.

The anti-obesity potential of arctigenin has also been demonstrated in several studies, as indicated by decreased levels of subcutaneous fat, serum cholesterol, and muscle triglyceride in obese mouse models^16^, possibly through the activation of AMP-activated protein kinase (AMPK)^16,17^. Therefore we investigated in the present study the ability of arctigenin to inhibit prostate cancer growth in obese conditions both *in vitro* and *in vivo*. Results from this study demonstrate that in addition to the inhibition of androgen receptor (AR), the key modulator of prostate tumor growth, arctigenin was able to increase the expression of Nkx3.1 and suppress tumor angiogenesis to inhibit prostate tumor growth in obese conditions. Nkx3.1 is a prostate specific homeobox gene and a tumor suppressor^18^. Loss of Nkx3.1 expression is widely observed in prostate cancer associated with cancer progression^18,19^. Angiogenesis is the formation of new blood vessels from preexisting ones, which plays a vital role in solid tumor growth^20,21^. Many factors are involved in angiogenesis, including vascular endothelial growth factor (VEGF) which is identified as a key driver of angiogenesis, insulin-like growth factor (IGF)-1, and monocyte chemoattractant protein (MCP)-1^22–24^.

## Results

### *In vitro* anti-proliferative effect and signaling regulation by arctigenin

**Figure 1:**
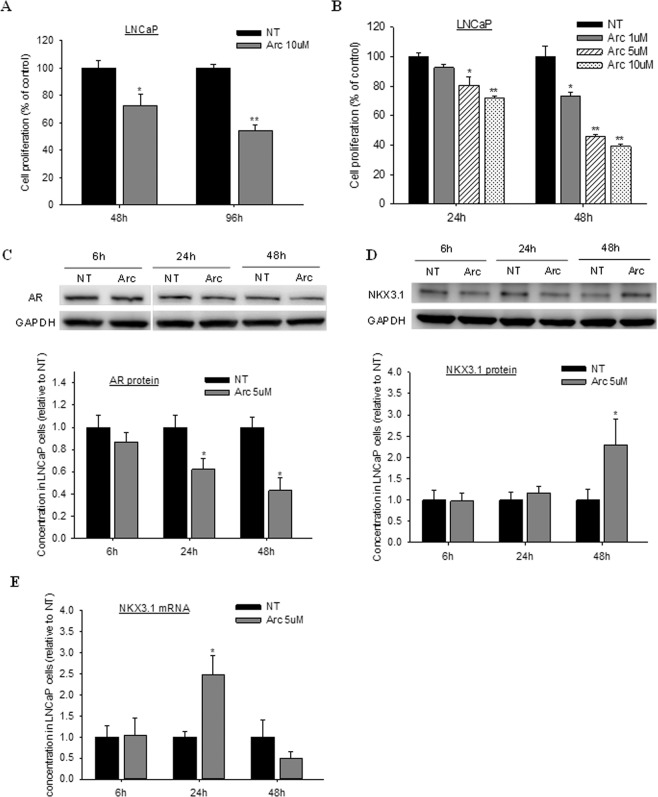
*In vitro* anti-proliferative effect and signaling regulation of arctigenin in prostate cancer cells. Androgen-sensitive human prostate cancer LNCaP cells were either co-cultured with mouse adipocytes 3T3-L1 (**A**) or cultured in adipocytes-conditioned medium (**B**–**E**), and treated with arctigenin over different time points. Cell proliferation was measured by adenosine triphosphate (ATP) assay, protein expression by Western blot, and mRNA expression by quantitative real-time PCR analysis. Cropped gels/blots are displayed, with full-length/uncropped gels and blots included in Supplementary Information file. Data are presented as mean ± SD. NT: non-treatment, DMSO control; Arc: arctigenin. Compared to NT, ^*^P < 0.05, ^**^P < 0.01.

Culture of LNCaP cells in obese conditions significantly enhanced cell proliferation compared to regular medium (Supp. Fig. 1). The anti-proliferative effect of arctigenin was assessed first in an *in vitro* obese model by the co-culture of LNCaP cells and 3T3-L1 adipocytes. Arctigenin treatment at 10 µM significantly inhibited LNCaP cell proliferation after 48 h incubation, with an inhibition by 50% at 96 h (Fig. 1A). Analysis of 3T3-L1 conditioned medium revealed that the levels of three adipokines/cytokines, including IGF-1, VEGF, and MCP-1, were significantly elevated relative to blank medium (Supp. Fig. 2A). The treatment with arctigenin significantly decreased both IGF-1 and VEGF levels in the co-culture medium as compared to control (Supp. Fig. 2B).

The second experiment was performed using 3T3-L1 adipocytes-conditioned medium for culture of LNCaP cells. Consistent to the first experiment, the proliferation of LNCaP cells was significantly inhibited by arctigenin treatment in a time- and dose-dependent manner (Fig. 1B). Western blot analysis showed that the protein expression of AR in LNCaP cells was significantly reduced by arctigenin after 24 h treatment (Fig. 1C), along with an increased protein expression of Nkx3.1 at 48 h (Fig. 1D). RT-PCR analysis demonstrated a significant increase in Nkx3.1 mRNA expression at 24 h of arctigenin treatment (Fig. 1E).

### Tumor growth inhibition and blood/tumor concentrations of arctigenin

**Figure 2:**
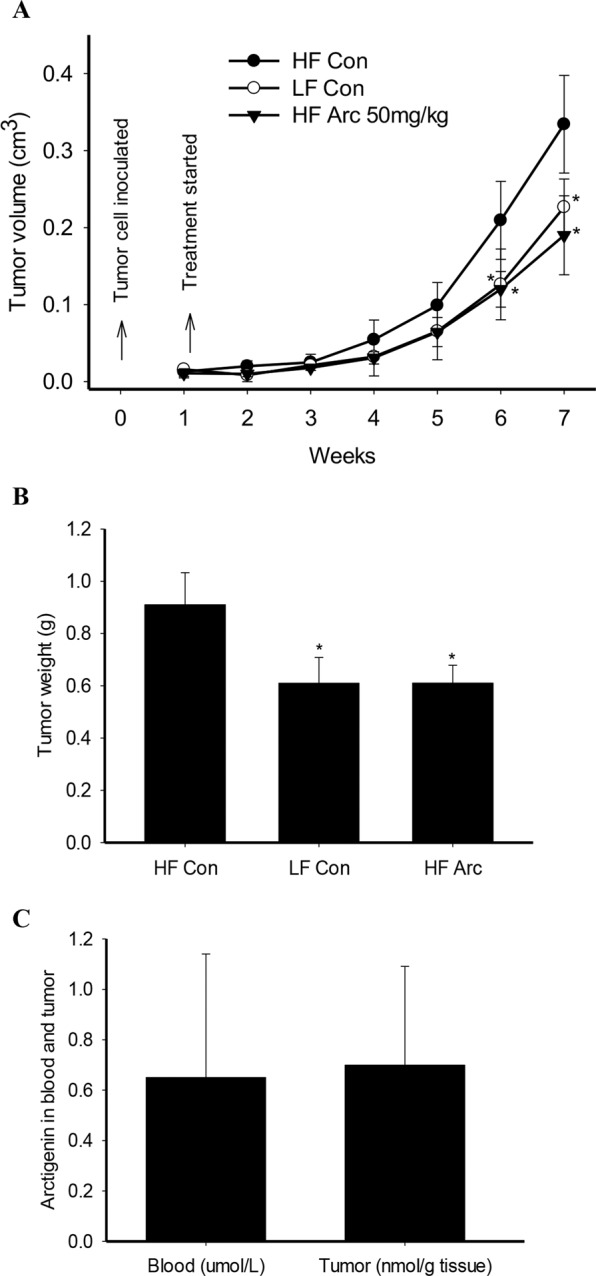
Arctigenin inhibits xenograft prostate tumor growth in high-fat diet fed SCID mice. Male SCID mice were fed either a high-fat (HF) diet or a regular low-fat (LF) diet (n = 10 per group). One week later, mice were inoculated subcutaneously with 5 × 10^5^ androgen-sensitive human prostate cancer LAPC-4 cells. The intervention started one week post tumor cell inoculation when tumors reached a volume of around 10 mm^3^. Mice were treated with either arctigenin at 50 mg/kg or vehicle control. Tumor size was measured once a week using calipers and tumor volume (**A**) calculated using the formula: length × width × height × 0.5236. Tumor weight (**B**) was measured at mouse sacrifice. Arctigenin was detected in blood and tumor from arctigenin supplemented mice (**C**) with HPLC coupled with CoulArray electrochemical detector after extraction with ethyl acetate. Data are presented as mean ± SD. Con: vehicle control; Arc: arctigenin. *Compared to control, P < 0.05.

To investigate the effect of different diets on tumor growth, mice of xenograft prostate tumors were fed either high-fat (HF) diet or regular low-fat (LF) diet for 6 weeks. The consumption of HF diet significantly increased tumor growth as compared to LF diet (Fig. 2A). Arctigenin intervention in mice on HF diet slowed down tumor growth as compared to HF control (Fig. 2A). Tumor growth was significantly inhibited by 45% after 5-weeks arctigenin intervention compared to HF control (Fig. 2A). Tumor weight at sacrifice showed consistent pattern with that of tumor volume (Fig. 2B).

Arctigenin was undetectable in the HF control group, while detectable in all the blood and tumor samples from the HF arctigenin group. The blood concentration of arctigenin was 0.65 ± 0.49 µmol/L, and tumor concentration 0.70 ± 0.39 nmol/g tumor (Fig. 2C), with a significant correlation between blood and tumor concentrations (*r = *0.98, P < 0.01).

### Reduced tumor proliferation marker and microvessel density

**Figure 3:**
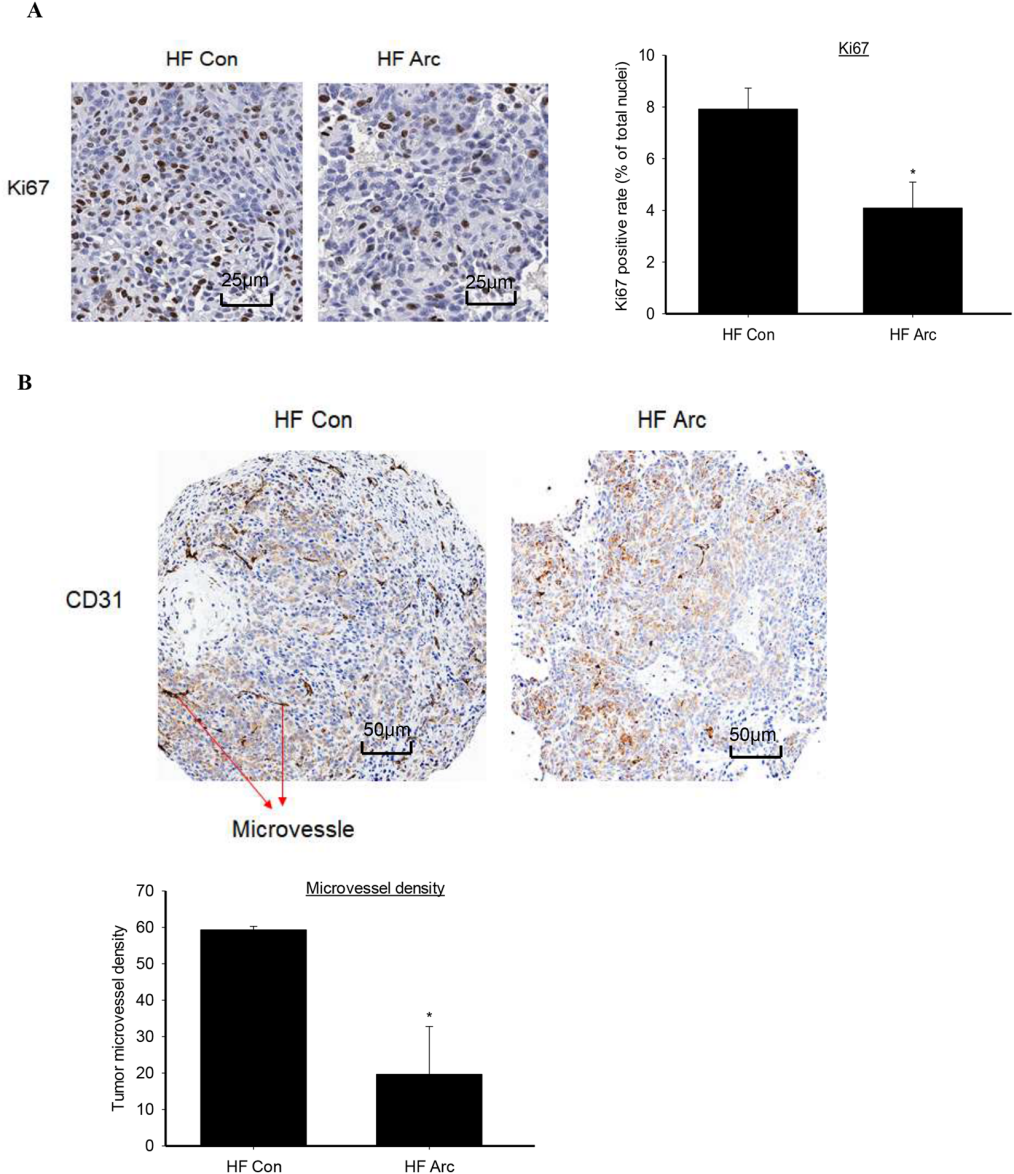
Arctigenin reduced tumor proliferation and microvessel density. (**A**) Shows the representative images of immunohistochemical staining of proliferation marker Ki67 in tumor tissues from high-fat (HF) diet-fed mice. (**B**) Shows the staining of blood vessel marker CD31 for microvessel density evaluation in the tumor tissues. Slides were counterstained with hematoxylin. Nuclei were stained in blue and Ki67 or CD31 in brown color. The positive rates of Ki67 nuclear staining, and microvessel density are presented as mean ± SD. Con: vehicle control; Arc: arctigenin. *Compared to control, ^*^P < 0.05. (**A**) scale bar = 25 µm, x400 magnification; (**B**) scale bar = 50 µM, x200 magnification.

Immunohistochemical analysis of tumor tissues showed that the proliferation marker nuclear Ki67 protein levels were significantly reduced by 50% in arctigenin group compared to HF control (Fig. 3A). In addition, regarding the important role of angiogenesis in tumor development and progression, we evaluated the anti-angiogenic ability of arctigenin in this study. CD31, also known as platelet endothelial cell adhesion molecule-1 (PECAM-1), is a membrane glycoprotein highly expressed in early and mature endothelial cells, and commonly used as a staining marker for microvessels in evaluation of tumor angiogenesis^25,26^. A strong inhibition of tumor angiogenesis was observed with arctigenin treatment, as indicated by a 70% reduction of total microvessel density, as well as the density of microvessels with lumen, in tumor tissues of arctigenin group as compared to HF control (Fig. 3B).

### Modulation of signaling molecules

**Figure 4:**
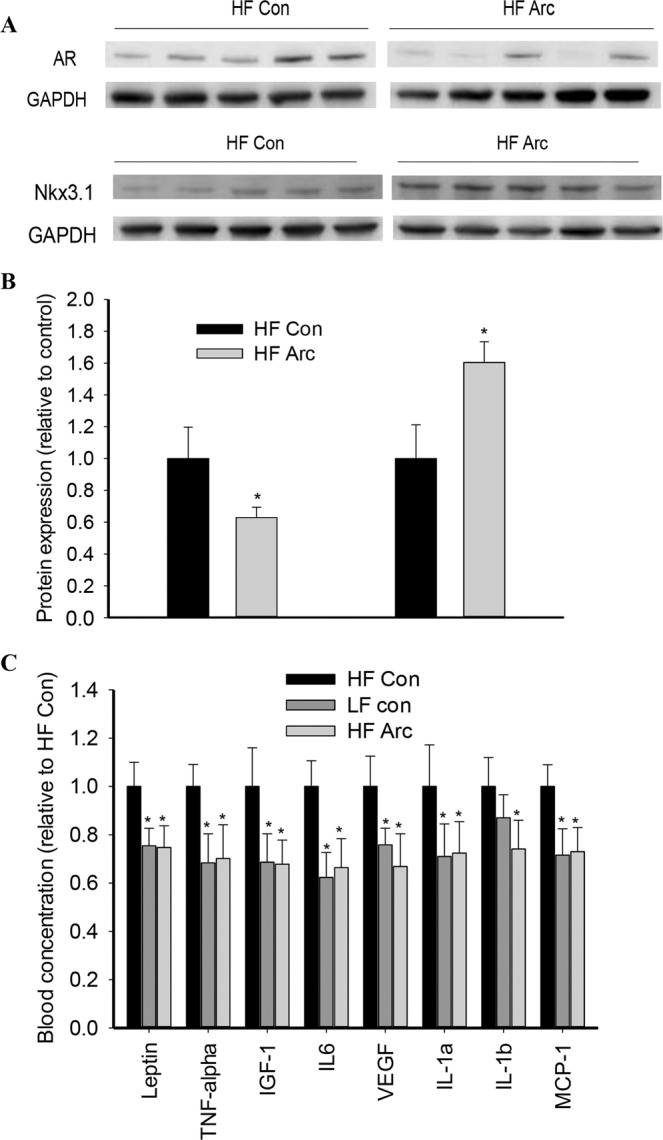
Modulatory effect of arctigenin on tumor signaling and adipocyte secretion of cytokines. Total protein was extracted from tumor tissues of high fat (HF) diet-fed mice for Western blot analysis of AR and Nkx3.1 expression (**A**) and quantification (**B**). Cropped blots are displayed, with uncropped gels included in Supplementary Information File. Five representative sample images are presented. The blood concentrations of 8 obesity-related growth factors (**C**) were measured using a Mouse Obesity ELISA Strip kit (Signosis, inc., Santa Clara, CA). Con: vehicle control; Arc: arctigenin. *Compared to control, *P < 0.05.

Western blot analysis of important signaling molecules involved in prostate cancer demonstrated that the AR protein expression was significantly reduced by arctigenin treatment compared to HF control, along with a significantly increased protein expression of Nkx3.1 (Fig. 4A,B). The blood concentrations of several major obesity-related adipokines/cytokines were significantly elevated in HF control mice compared to that in LF control (Fig. 4C). Arctigenin treatment significantly decreased the blood levels of leptin, tumor necrosis factor (TNF)-α, IGF-1, interleukin (IL)-6, VEGF, IL-1a, IL-1b, and MCP-1 by 30–35% compared to HF control (Fig. 4C).

### Metabolic regulations by arctigenin

**Figure 5:**
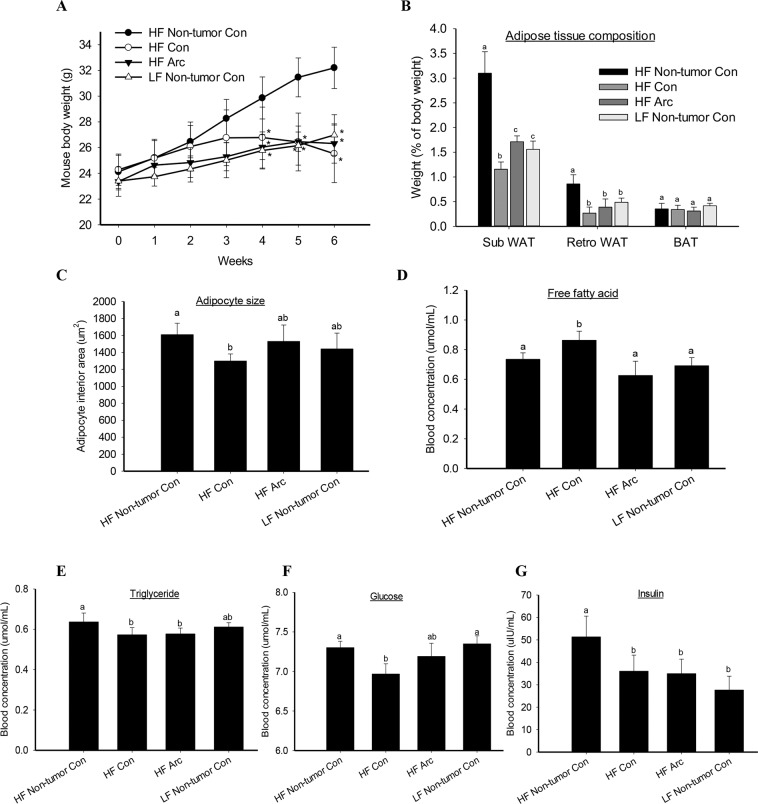
Metabolic regulations of arctigenin on adipose tissue. Mouse body weights (**A**) were measured over the intervention. Adipose tissues (**B**) were weighed at sacrifice, and a section of subcutaneous white adipose tissue (WAT) was fixed in 10% phosphate buffered formalin and paraffin-embedded for adipocyte size measurement (**C**) using a Definiens’ Tissue Studio. Blood samples were used for analysis of free fatty acid (**D**) with a Free Fatty Acid Quantification Kit (Abcam, Cambridge, MA), triglyceride (**E**) with a PicoProbe Triglyceride Quantification Assay Kit (Abcam), cholesterol with an HDL and LDL/VLDL Cholesterol Assay Kit (Abcam), glucose (**F**) with a glucose Detection Kit II (Abcam), and insulin (**G**) with an Insulin ELISA Kit (ThermoFisher Scientific, Waltham, MA), following the manufacturers’ instructions. HF: high fat diet; LF: low fat diet; Con: vehicle control; Arc: arctigenin. Sub WAT: subcutaneous white adipose tissue; retro WAT: retroperitoneal white adipose tissue; BAT: brown adipose tissue. *Compared to control, *P < 0.05. Columns with different letters indicate significant difference between groups, P < 0.05.

There was no significant difference in mouse body weight between the arctigenin and control groups on HF diet (Fig. 5A). However, as compared to the non-tumor control mice that were fed high-fat diet (HF Non-tumor Con), mice bearing tumors had significantly reduced body weights after 4 weeks of tumor cell inoculation (Fig. 5A). Body weights in HF arctigenin group were comparable to that of LF non-tumor bearing mice, while body weights of HF control mice showed a slight trend while not statistically significant decrease starting 3 weeks post tumor inoculation (Fig. 5A). The weight of white adipose tissue (WAT), particularly the subcutaneous WAT, was proportional to mouse body weight (r = 0.62, P < 0.05), with a significantly reduced level in the tumor-bearing HF control mice compared to other groups (Fig. 5B). There was no difference in brown adipose tissue weight among groups (Fig. 5B). Consistent with our observation of reduced subcutaneous WAT mass in HF control mice, there was a significant reduction in adipocyte size of the subcutaneous WAT in HF control group compared to other groups (Fig. 5C).

Blood FFA, which is mainly released from adipose tissue, is an important source of fatty acid for tumor cell proliferation *in vivo*^27^. In this study we found that serum FFA levels were significantly higher in the tumor-bearing HF control mice compared to non-tumor mice and arctigenin-treated HF mice (Fig. 5D). There was no difference in blood triglyceride levels between HF arctigenin and HF control groups, while both groups had lower blood triglyceride levels as compared to HF non-tumor mice (Fig. 5E). There was no difference in blood cholesterol levels among the four groups (data not shown). The blood glucose levels in HF control mice were significantly lower as compared to non-tumor control mice, while the levels in HF arctigenin-treated mice were comparable to that in non-tumor mice (Fig. 5F). The blood insulin levels were higher in HF non-tumor mice compared to other groups, while there was no difference between HF arctigenin and HF control groups (Fig. 5G).

## Discussion

We demonstrate in this study that arctigenin, at relatively low doses, significantly inhibited prostate cancer growth in obese conditions both *in vitro* and *in vivo*. Obesity elevates circulating levels of multiple growth factors and pro-inflammatory cytokines/chemokines, such as IGF-1, VEGF and MCP-1 among others, leading to accelerated tumor growth as demonstrated in this study and by other investigators^5,28^. In addition, obesity contributes to the failure of cancer treatments due to increased inflammation, metabolic perturbation, and induction of chemoresistance^8^. Therefore, it is critical to co-target obesity in prevention and treatment of cancer particularly in obese patients.

We previously demonstrated a strong anti-tumor effect of arctigenin in the non-obese state with an IC_50_ value of 2 µM in LNCaP cells, which is about 10–20 fold stronger than most of phytochemicals being studied including green tea polyphenols^15,29^. The culture of LNCaP cells with adipocytes or with adipocytes-conditioned medium in the present study moderately increased the IC_50_ of arctigenin to 5–10 µM, at least partly due to the adipocytes’ secretion of multiple growth factors such as IGF-1, VEGF and MCP-1 into the co-culture medium. Our *in vivo* study further confirmed the anti-tumor effect of arctigenin in obese conditions by showing a 45% inhibition of prostate tumor growth, along with significantly reduced tumor expression of the proliferation marker Ki67. The *in vivo* anti-tumor potency of arctigenin as demonstrated in this study was close to our previous observations in LF diet fed SCID mice where arctigenin inhibited xenograft prostate tumor growth by 50% at the same dose level (equivalent to 300 mg arctigenin daily for an average adult)^15^. These results support arctigenin to be a strong anticancer agent in both non-obese and obese states. The blood concentration of arctigenin in this study was similar to that we observed previously in the LF diet fed mice (0.62 ± 0.07 μmol/L), while the tumor concentration of arctigenin in this study was lower than that in the LF diet fed mice (4.7 ± 3.3 nmol/g) in our previous study^15^, which may suggest a reduced cellular uptake of arctigenin in the relatively nutrient (fatty acids)-rich environment in this study.

Our mechanistic investigations revealed that the reduced tumor growth by arctigenin treatment was associated with a reduction of microvessel density in tumors. Angiogenesis is critically important for the growth of solid tumors, and tumors will remain in dormant phase for a long time in absence of blood vessel formation^20,21^. The anti-angiogenic activity of arctigenin was also observed in previous studies associated with the inhibition of tumor growth and invasion^30,31^. Tumor angiogenesis is significantly enhanced in obese state, mainly due to the elevated circulating pro-angiogenic/-inflammatory factors released from adipocytes, such as VEGF, IGF-1, MCP-1, IL-1, TNF, etc^24^. Results from this study demonstrate the ability of arctigenin to regulate the expression of these pro-angiogenic/-inflammatory factors, leading to a strong inhibition of tumor angiogenesis in the studied stimulatory environment.

The ability of arctigenin to metabolically regulate supplies of cancer nutrients such as fatty acids as demonstrated in this study may contribute to its anti-tumor effect. Fatty acids are essential for cancer cell proliferation by providing building blocks for synthesis of membranes and signaling molecules as well as supply of energy^32^. Circulating free fatty acids released from adipocytes are the major supply of fatty acids to tumor cells, and limiting the availability of fatty acids to cancer cells have shown to be effective in inhibition of proliferation of different cancer cells^32,33^. In the present study, we observed that blood concentrations of free fatty acids in HF control mice were even higher than that in HF non-tumor mice, which is opposite to their body weights or adipose tissue weights. This suggests that cancer cells may be able to induce the release of free fatty acid from adipocytes to satisfy their growth need, and this may eventually lead to a state of cachexia as a sign of which in this study was a decreasing trend in body weights of HF control mice after three weeks of tumor growth. Elevated blood free fatty acid as a result of elevated lipolysis due to increased energy expenditure was also observed in previous studies at early stage of cancer cachexia^34,35^. A decrease in blood glucose level due to tumor presence was also reported in previous studies, associated with loss in body weight and fat weight as a result of increased energy expenditure by tumor growth^36,37^. However, blood glucose levels were still within normal values in many patients of cachexia, although blood insulin levels could be high, normal or low in correlation with degree of weight loss^38^. Blood triglycerides mainly come from dietary intake, and previous studies found that blood triglyceride levels were irrespective of the extent of weight loss in cancer cachexia^39^. In our study the mice were fed high-fat diet and the food consumption was similar in HF control and HF Arc groups, which may explain the similarity of blood triglyceride levels observed between these two groups. Arctigenin was able to significantly reduce the circulating free fatty acid levels compare to HF control, and to maintain mouse body weight and blood free fatty acid and glucose at levels comparable to that in LF non-tumor mice. These data suggest the strength of arctigenin in regulation of the systemic environment to control external tumor-promoting factors in inhibition of tumor growth.

The direct regulation of tumor cell signaling is involved in the mechanisms of arctigenin in tumor inhibition. We previously found that arctigenin is a strong inhibitor of the AR signaling in non-obese state both *in vitro* and *in vivo*, while it is a stimulator of the Nkx3.1 signaling in prostate tumor cells^15,40^. The present study further demonstrates the association of AR and Nkx3.1 with the tumor inhibitory effect of arctigenin in obese conditions. AR is a key modulator of proliferation and progression of prostate cancer, which makes it an important target in prostate cancer chemoprevention and treatment^41^. AR has also been found to be positively associated with VEGF levels in human prostate tumors^42^, partly through the activation of hypoxia-inducible factor 1 (HIF-1)^43^. Therefore the reduced angiogenesis in the present study may be partly due to the suppressed AR expression by arctigenin treatment. Nkx3.1 is found to be a prostate tumor suppressor, and loss of Nkx3.1 protein expression is commonly observed in prostate tumors^19^. The increased Nkx3.1 expression under arctigenin treatment in this study was concurrent with the decrease in AR expression, and may contribute to the tumor-inhibitory effect of arctigenin. These data support the strong capacity of arctigenin in regulating multiple aberrant tumor signaling pathways in control of tumor growth.

In summary, this study demonstrates the strength of arctigenin in inhibition of prostate cancer growth both *in vitro* and *in vivo* in obese conditions. The anti-tumor effect of arctigenin was associated with its dual actions on both tumor itself to control dysregulated tumor signaling like AR and Nkx3.1 and on adipose tissues to reduce the secretion of pro-cancer adipokines/cytokines as well as the release of free fatty acid. Considering the challenge greatly increased by obesity in treatment of cancers, this study is anticipated to bring significant benefits in treatment of prostate cancer particularly in obese patients by providing a promising agent to co-target obesity and cancer in a non-toxic manner.

## Methods

### Cell culture and adipocyte differentiation

Androgen-sensitive LNCaP and LAPC-4 human prostate cancer cell lines were purchased from ATCC (Chicago, IL, USA), maintained in RPMI 1640 medium supplemented with 10% (v:v) of fetal bovine serum (FBS), 100 IU/mL of penicillin and 100 µg/mL of streptomycin at 37 °C in a 5% CO_2_ incubator. 3T3-L1 mouse embryonic fibroblasts (ATCC) were cultured in Dulbecco’s modified Eagle’s medium supplanted with 10% FBS, 100 IU/mL of penicillin and 100 µg/mL of streptomycin. Differentiation of 3T3-L1 into adipocytes was performed as described previously with minor modifications^44^. Briefly, 3T3-L1 cells were grown into confluence and incubated as confluent culture for another 48 h. Cells were then treated with differentiation medium composed of growth medium plus 1.0 μM dexamethasone, 0.5 mM methylisobutylxanthine, and 1.0 μg/mL Insulin (all from Sigma-Aldrich, St. Louis, MO). Cells were incubated for 12 days in differentiation medium with fresh medium replaced every 48 h. The full differentiation was evidenced by observation of lipid droplet formation by light microscopy, and the differentiated adipocytes were maintained in growth medium. All cell lines were tested periodically for mycoplasma contamination. Passages of cell lines less than 10 were used for this study.

### *In vitro* cell proliferation assay

In the first experiment, an *in vitro* obesity model was constructed by co-culturing of LNCaP cells and 3T3-L1 adipocytes in a 24-well plate. Adipocytes were seeded on the bottom of wells at a density of 80 cells per µL in 600 µL RPMI medium 24 h prior to LNCaP seeding on inserts. Adipocytes-conditioned medium was collected on the day of LNCaP seeding and mixed with fresh RPMI medium at 3:7 ratio (v:v) as co-culture medium. LNCaP cells were seeded at 80 cells/uL on the Falcon Cell Culture Inserts with a pore size of 0.4 μM (Fisher Scientific). Arctigenin treatment at 10 µM or DMSO control was added to the co-culture medium. Fresh co-culture medium plus treatments was replaced at 48 h for the 96 h time point. To detect cell proliferation, the inserts with LNCaP cells were transferred to a clean 24-well plate and adenosine triphosphate (ATP) assay reagent from the CellTiter-Glo Luminescent cell viability assay kit (Promega Corporation, Madison, WI) was added to each insert following the manufacturer’s instruction. After incubation, 200 uL of supernatant was transferred to an opaque wall 96-well plates for chemiluminescence detection.

In the second experiments, 3T3-L1 adipocytes-conditioned medium was generated. 3T3-L1 adipocytes were seeded in T75 flasks at a density of 100 cells per µL in 10 ml RPMI medium. After 48 hours, medium was changed to fresh medium. After another 24 hours, the medium was collected, centrifuged at 2,000 rpm for 10 min, and supernatant transferred to a new tube as conditioned medium. The conditioned medium was mixed with fresh RPMI medium at 1:1 ratio for culture of LNCaP cells in a 96-well plate. Cells were treated with 1, 5, or 10 µM of arctigenin or DMSO control for 24 h and 48 h. Cell proliferation was measured using the ATP assay kit. Both experiments were performed in quadruplicate and repeated twice.

### Animal study

All procedures carried out in mice were approved by the Institutional Animal Care and Use Committee at Charles R. Drew University of Medicine and Science (protocol # I-1606-277). All methods were performed in accordance with the relevant guidelines and regulations. Male severe combined immunodeficiency (SCID) mice at age of 5–7 weeks (Charles River Laboratories) were acclimatized on sterilized AIN-93G diet (Dyets Inc., Bethlehem, PA) and water for one week. Mice were then fed a HF diet containing 45% energy from fat (Research Diets, Inc., New Brunswick, NJ) until the end of the study. One week after the start of HF diet, mice were inoculated subcutaneously with 5 × 10^5^ androgen-sensitive LAPC-4 human prostate cancer cells. The intervention started one week later when tumors reached a volume of around 10 mm^3^. Mice were randomly assigned to receive arctigenin at 50 mg/kg body weight (b.w.) daily or vehicle control (2% DMSO in corn oil) through oral gavage, with 10 mice in each group. An additional control group (n = 10) fed regular LF diet containing 10% energy from fat (Research Diets) was added in parallel for comparison of the diet effect on tumor growth. Tumor size was measured once a week using calipers. Tumor volume was calculated with the formula: length × width × height × 0.5236^45^. Mouse body weight was measured once a week. The intervention lasted for 6-weeks. In addition, two groups of SCID mice without tumor inoculation were included on either HF diet or LF diet (n = 10 per group) for comparison of metabolic parameters and body weights.

### Western blot analysis of protein marker expression

In the *in vitro* study, LNCaP cells were cultured in medium containing 3T3-L1 adipocytes-conditioned medium plus fresh RPMI medium at 1:1 ratio, and treated with 5 µM of arctigenin or DMSO control for 6 h, 24 h, and 48 h. Total protein was extracted from the cells for Western blot analysis of AR and Nkx3.1 expression following procedures as described previously^46,47^. Three biologically independent experiments were performed, and each protein sample was detected in duplicate in Western blot analysis. Mean values and standard deviations were calculated from the total 6 values of each sample. Total protein extracted from tumor tissues of the mouse studies (n = 10 mice per group, each sample tested in duplicate) was also measured for AR and Nkx3.1 expression. Membranes were incubated with primary antibody for detection of AR (sc-7305) or Nkx3.1 (sc-15022, Santa Cruz, CA). GAPDH protein was used as loading control.

### Quantitative real-time PCR analysis of Nkx3.1 gene expression

LNCaP cells were treated the same as above for western blot analysis. Total RNA was extracted for quantitative real-time PCR analysis following previous procedures^47,48^. The final volume of the 20 μL real-time PCR mixture contained 5 μL of cDNA template, 1 μL of each primer, 10 μL of SYBR Green (Qiagen, Valencia, CA), and 3 μL of nuclease-free water. Each sample was measured in triplicate. The 2^−(∆∆Ct)^ method was used to normalize the expression of Nkx3.1 to GAPDH and to compare the average ∆Ct value. The primers for Nkx3.1 included the forward 5′-GAA TCC GTA TGC CCC GCT GAA TCT-3′ and reverse 5′-ACC CTT GCC AGT GCG TGT GC-3′. Primers for GAPDH include the forward 5′-CAT GTT CGT CAT GGG TGT GA-3′ and reverse 5′-GGT GCT AAG CAG TTG GTG GT-3′. Three biologically independent experiments were performed, and each sample was measured in triplicate in PCR analysis.

### HPLC detection of arctigenin in mouse blood and tumor tissues

Blood (n = 10 in each group) and tumor (n = 10 in each group) samples collected from mice on HF diet, including the control and arctigenin groups, were measured for arctigenin concentrations following a previous method^15,46^. Briefly, 200 µl of serum was extracted twice with 1 mL of ethyl acetate each time. For tumors, about 150 mg of tumor tissue was homogenized in 200 µl of water containing 2% ascorbic acid, and extracted twice with 1 mL of ethyl acetate each time. The supernatant was pooled, dried under nitrogen flow and reconstituted for high-performance liquid chromatography (HPLC) detection coupled with a CoulArray electrochemical detector (ThermoFisher Scientific, Madison WI).

### Immunohistochemical analysis of tumor Ki67 expression and microvessel density

A section of each tumor (n = 10 in each group) from mice on HF diet, including the control and arctigenin groups, was fixed in 10% phosphate buffered formalin and paraffin-embedded for tissue microarray and immunohistochemical detection as described previously^49^. The tissue array was assembled with 6 cylindrical cores from each tumor. Slides were incubated with monoclonal anti-human Ki67 or anti-mouse CD31 antibody for microvessel (DAKO North America Inc., Carpineteria, CA), and counterstained with hematoxylin. Slides were scanned on a ScanScope AT (Aperio Technologies, Inc., Vista, CA) and morphometric analysis performed digitally with Definiens’ Tissue Studio (Definiens Inc., Parsippany, NJ) in a non-biased method. Briefly, the nuclear detection module and classification tool were pre-defined to identify positive and negative nuclei within each core. Thresholds were set to classify negative nuclei with hematoxylin stain and positive nuclei with DAB stain. Data were exported to Excel for statistical analysis. Scanning and analyses were performed at the UCLA Department of Pathology.

### Measurement of obesity-related growth factors

The concentrations of 8 obesity-related growth factors in cell culture medium (three biologically independent experiments, each samples tested in duplicate) or in mouse blood (n = 10 in each group, each samples tested in duplicate) were measured using a Mouse Obesity ELISA Strip kit (Signosis, inc., Santa Clara, CA) following the manufacturer’s instruction. Briefly, 100 µL of cell culture medium or 100 µL of 1:10 diluted blood was loaded on the 8-well strip pre-coated with different antibodies, followed by addition of a biotin-labeled antibody and then streptavidin-HRP conjugate. After adding a HRP substrate the luminescence intensity was measured which is proportional to the concentrations of growth factors.

### Analysis of mouse adipocyte size

At sacrifice a section of subcutaneous WAT was fixed in 10% phosphate buffered formalin and paraffin-embedded. WAT samples from 10 mice in each group were assembled on a microarray block, with 3 cores from each sample. Slides were cut and H&E stained. Slides were digitized and adipocyte sizes measured using the Definiens’ Tissue Studio at the UCLA Department of Pathology. There were between 100 and 200 adipocytes on each core of the WAT. The average values of adipocyte size from 3 cores of the same sample/mouse were calculated (10 average values in total). The mean values and standard deviations of adipocyte size in each group were calculated using the 10 average values.

### Measurement of mouse blood lipid, cholesterol, glucose, and insulin

The concentrations of free fatty acid in mouse blood (n = 10 in each group, each samples tested in duplicate) were measured with a Free Fatty Acid Quantification Kit (Abcam, Cambridge, MA), triglyceride with a PicoProbe Triglyceride Quantification Assay Kit (Abcam), cholesterol with an HDL and LDL/VLDL Cholesterol Assay Kit (Abcam), glucose with a glucose Detection Kit II (Abcam), insulin with an Insulin ELISA Kit (ThermoFisher Scientific, Waltham, MA), following the manufacturers’ instructions. A standard curve was made for each analyte to calibrate their concentrations in blood.

### Statistical analysis

SPSS software (Version 20, Chicago, IL) was used for statistical analysis. Mean values and standard deviation (SD) were calculated. Comparison of means was performed by two independent samples t-test or one-way analysis of variance (ANOVA) with Tukey’s posttest for paired comparison, or linear mixed-effects model for repeated measures. Correlation between mouse adipose tissue weight and body weight was analyzed by Pearson Correlation analysis. Differences were considered significant if P < 0.05.

## Supplementary information

Supplementary Information.
