# Supplementary material for: Arctigenin inhibits prostate tumor growth in high-fat diet fed mice through dual actions on adipose tissue and tumor
Source: Sci Rep. 2020 Jan 29;10:1403. doi: 10.1038/s41598-020-58354-3 (PMC6989655; doi:10.1038/s41598-020-58354-3)

Arctigenin inhibits prostate tumor growth in high-fat diet fed mice through dual actions on adipose tissue and tumor

Qiongyu Hao[1,5], Tanya Diaz[1], Alejandro del Rio Verduzco[1], Clara E. Magyar[2], Jin Zhong[6,7], Yahya Elshimali[1], Matthew B. Rettig[3], Susanne M. Henning[4], Jaydutt V. Vadgama[1,5], Piwen Wang[1,4, 5,*],

[1]Division of Cancer Research and Training, Charles R. Drew University of Medicine and Science, Los Angeles, CA, USA 90059; [2]Department of Pathology; [3]Departments of Medicine and Urology; [4]Center for Human Nutrition; [5]David Geffen School of Medicine, University of California, Los Angeles, CA, USA 90095; [6]VA Greater Los Angeles Healthcare System, Los Angeles, CA 90073; [7]University of California, Riverside, CA, USA 92521.

***Correspondence to:** Dr. Piwen Wang, Division of Cancer Research and Training, Charles R. Drew University of Medicine and Science, Los Angeles, CA 90059, USA. Tel: (323) 563-4999; Fax: (323) 563-4889; Email: piwenwang@cdrewu.edu

Supplemental Fig. 1

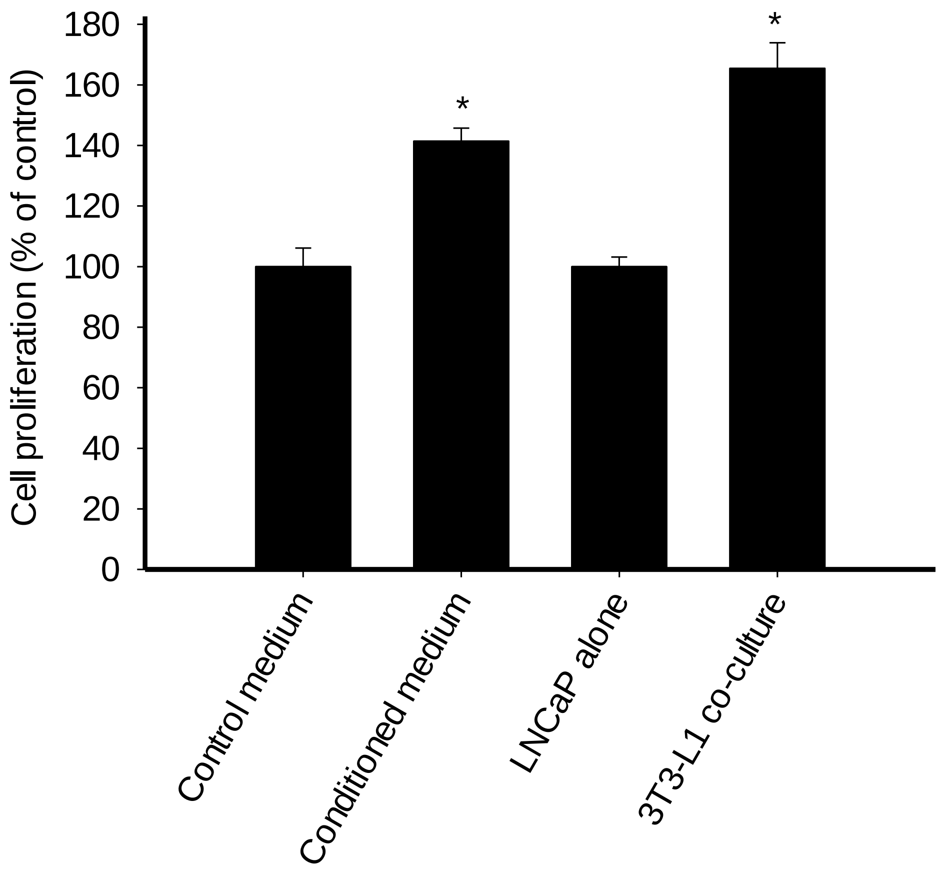

Supplemental Figure 1. Enhanced proliferation of LNCaP cells cultured with 3T3-L1 conditioned medium or co-cultured with 3T3-L1 adipocytes. LNCaP cells were cultured with complete RPMI-1640 (control medium), or 3T3-L1 conditioned medium mixed with fresh RPMI medium at 1:1 ratio. In the co-culture study, LNCaP cells were cultured on inserts alone or with pre-seeded 3T3-L1 cells on bottom. Cell proliferation was measured at 48h using ATP assay

Supplemental Fig. 2

A

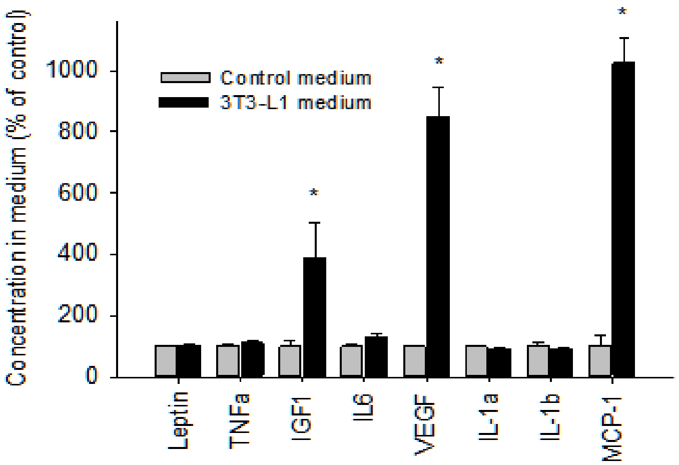

B

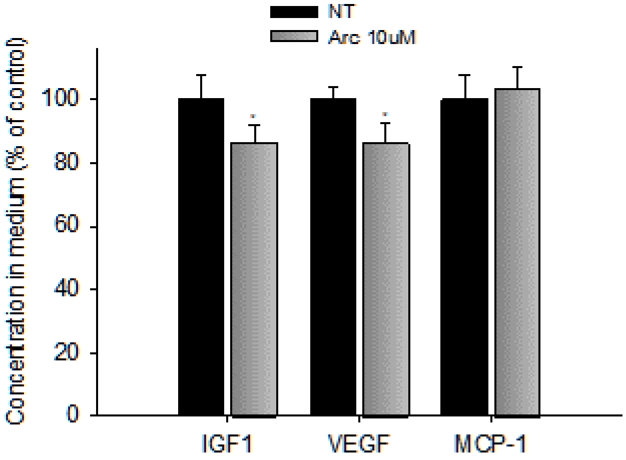

Supplemental Figure 2. Arctigenin inhibited the release of adipokines/cytokines from adipocytes. The concentrations of 8 obesity-related adipokines/cytockines in cell culture medium were measured using a Mouse Obesity ELISA Strip kit. The analysis of adipocytes 3T3-L1 conditioned medium was shown in A, and of the co-cultured medium under arctigenin treatment in B. NT: non-treatment, DMSO control; Arc: arctigenin. *Compared to NT, P<0.05.

Supplemental Figure 3. Full-length gels for Western blot data presented in Figure 1. A and B are gels for AR measurement. The first 2 lanes after ladder in A (6h) and the 3-6 lanes after ladder in B (24h and 48h) are presented in Figure 1C. The first 6 lanes after ladder in C are presented in Figure 1D. Densitometry data for individual bands are indicated.

A

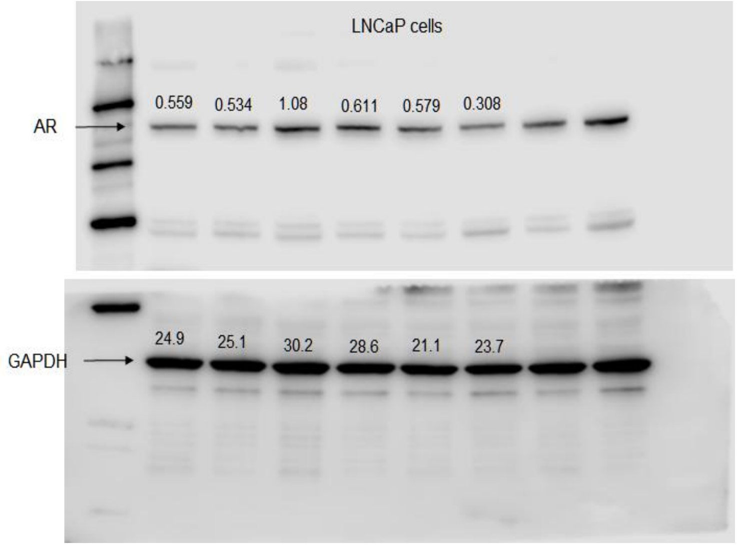

B

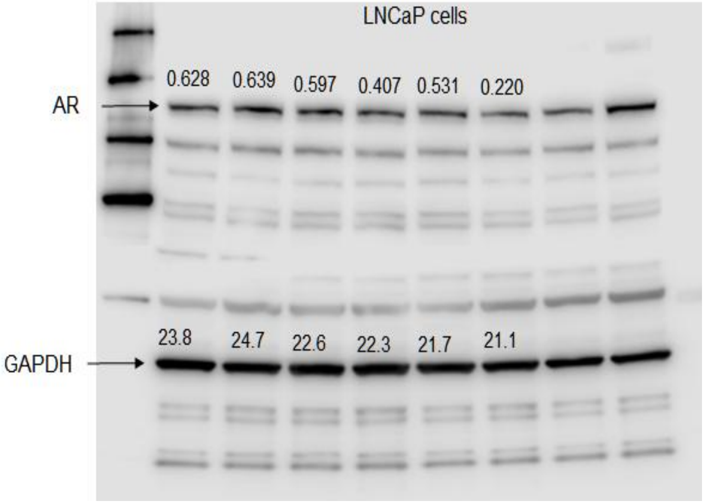

C

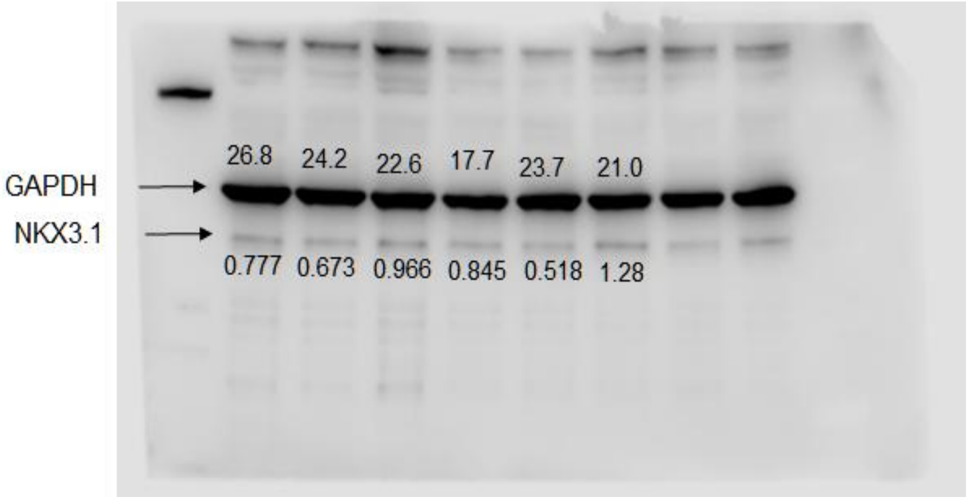

Supplemental Figure 4. Full-length gels for Western blot data presented in Figure 4 (last 5 samples are presented in Figure 4 for each group). Densitometry data for individual bands are indicated.

A. AR in HF Con group

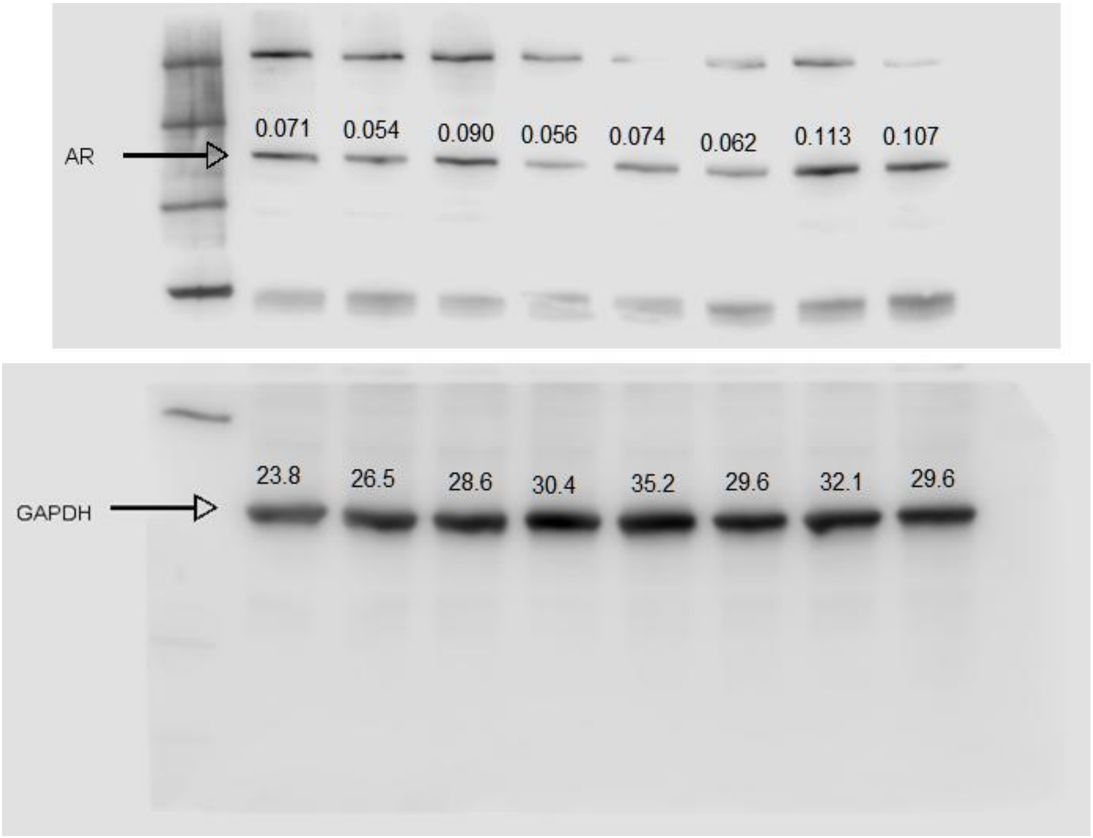

B. AR in HF Arc group

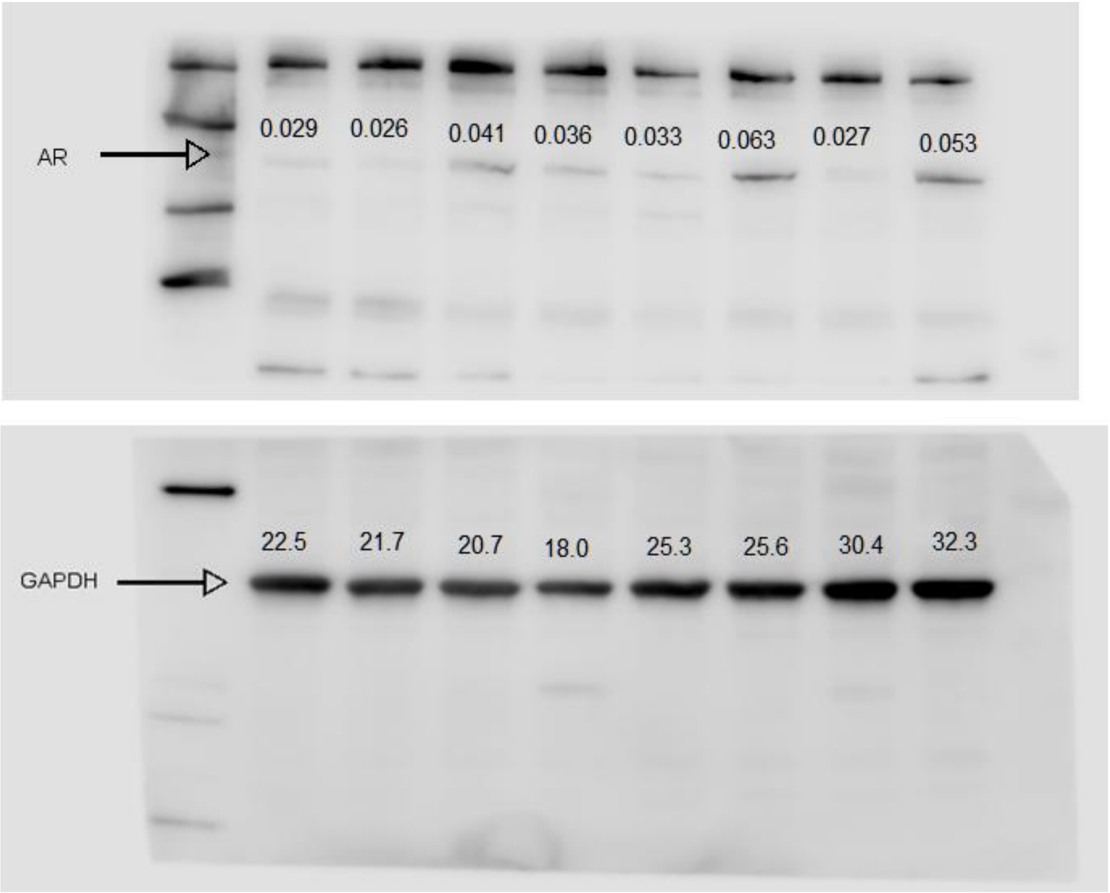

C. NKX3.1 in HF Con group

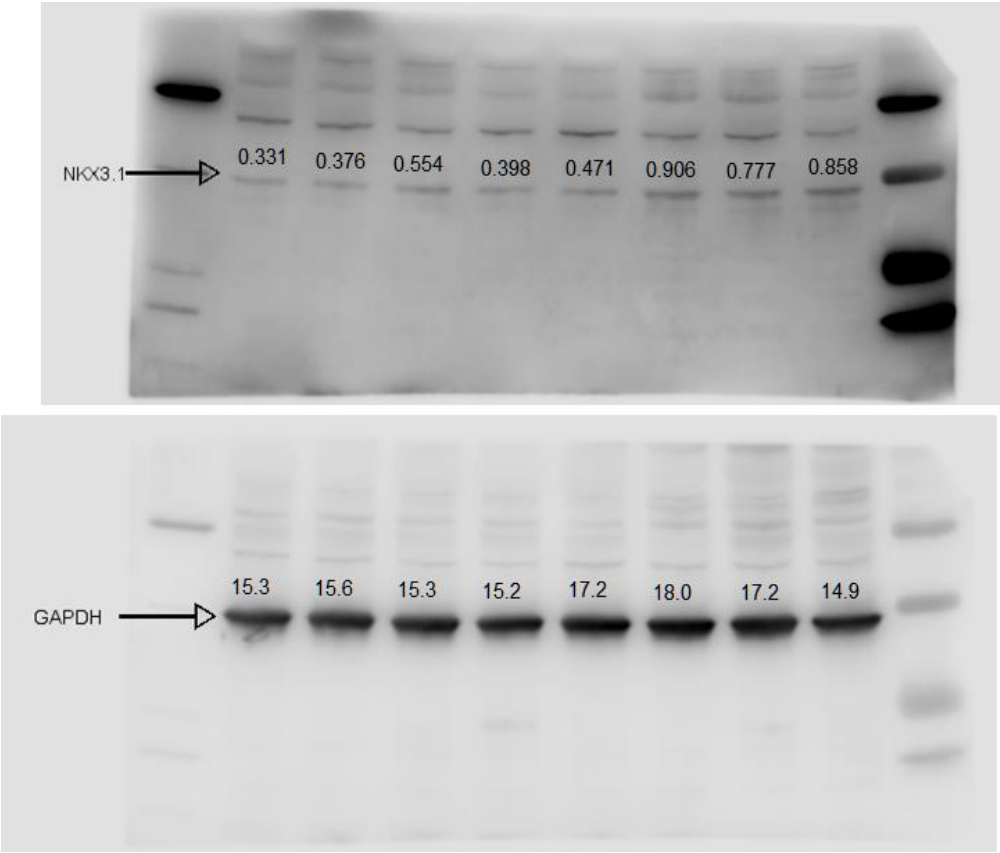

D. NKX3.1 in HF Arc group

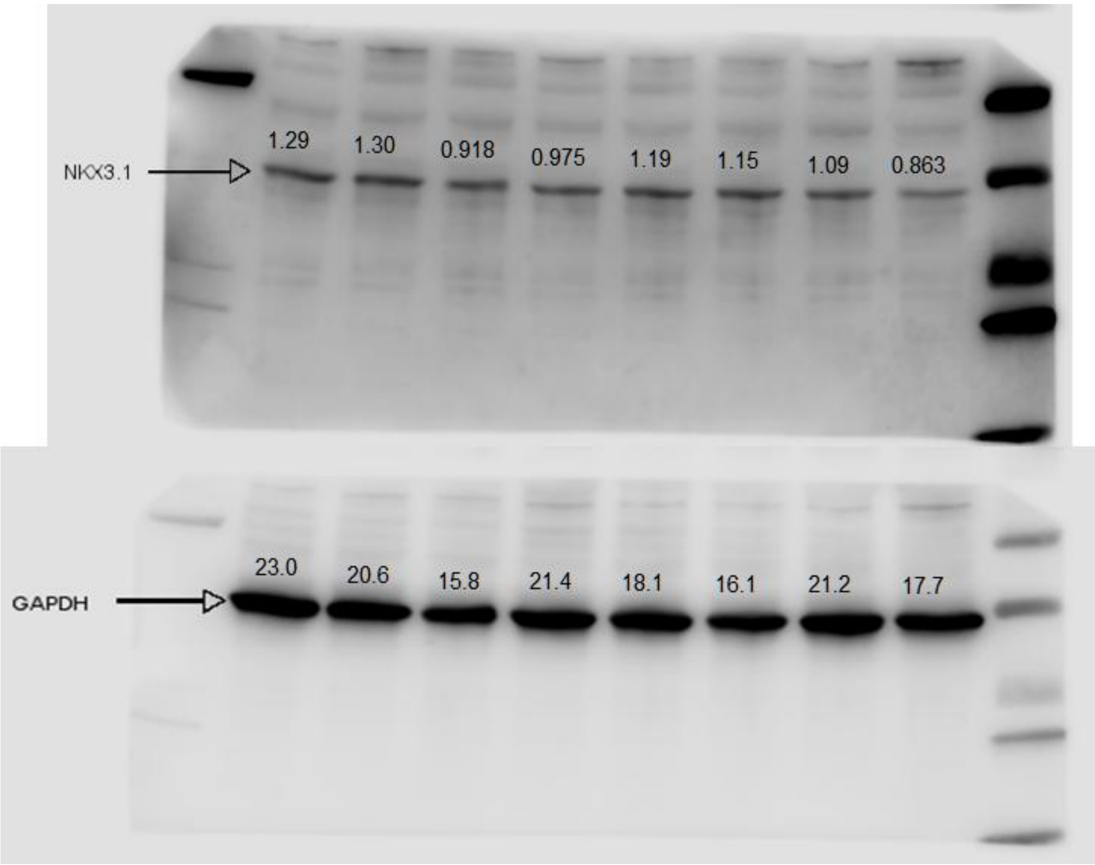

Supplement: Supplementary file 1 — Supplementary Information. [file 41598_2020_58354_MOESM1_ESM.pdf]
